# Horizontal equity in health care utilization in Brazil, 1998–2008

**DOI:** 10.1186/1475-9276-11-33

**Published:** 2012-06-21

**Authors:** James Macinko, Maria Fernanda Lima-Costa

**Affiliations:** 1 Department of Nutrition, Food Studies & Public Health, New York University, 411 Lafayette Street, 5th Floor, New York, NY, 10003, USA; 2 Centro de Pesquisas René Rachou, Fundação Oswaldo Cruz and School of Medicine, Federal University of Minas Gerais, Av. Augusto de Lima 1715, Belo Horizonte, CEP: 30190-002, MG, Brazil

**Keywords:** Healthcare, Brazil, Access to care, Primary care

## Abstract

**Introduction:**

This study assesses trends in horizontal equity in the utilization of healthcare services from 1998 to 2008--a period of major economic and social change in Brazil.

**Methods:**

Data are from nationally representative surveys repeated in 1998, 2003, and 2008. We apply established methods for assessing horizontal inequity in healthcare access (the principle that people with the same healthcare needs should have similar access to healthcare services). Horizontal inequity is calculated as the difference between observed healthcare utilization and utilization predicted by healthcare needs. Outcomes examined include the probability of a medical, dental, or hospital visit during the past 12 months; any health service use in the past two weeks; and having a usual source of healthcare. We use monthly family income to measure differences in socioeconomic position. Healthcare needs include age, sex, self-rated health, and chronic conditions. Non-need factors include income, education, geography, health insurance, and Family Health Strategy coverage.

**Results:**

The probability of having at least one doctor visit in the past 12 months became substantially more equitable over time, ending with a slightly pro-rich orientation in 2008. Any hospitalization in the past 12 months was found to be pro-poor in all periods but became slightly less so in 2008. Dental visits showed the largest absolute decrease in horizontal inequity, although they were still the most inequitably (pro-rich) distributed outcome in 2008. Service use in the past two weeks showed decreased inequity in 2003 but exhibited no significant change between 2003 and 2008. Having a usual source of care became less pro-rich over time and was nearly income-neutral by 2008. Factors associated with greater inequities include income, having a private health plan, and geographic location. Factors associated with greater equity included health needs, schooling, and enrolment in the Family Health Strategy.

**Conclusions:**

Healthcare utilization in Brazil appears to have become increasingly equitable over the past 10 years. Although this does not imply that equity in health outcomes has improved correspondingly, it does suggest that government policies aimed at increasing access, especially to primary care, have helped to make healthcare utilization in Brazil fairer over time.

## Introduction

There are considerable income disparities in Brazil, as reflected by one of the world’s highest Gini indices: 0.54 in 2009 [1]. There is also evidence of socioeconomic disparities in access to and use of healthcare [2,3]. Health disparities are particularly relevant as Brazil continues to develop its national health system (the *Sistema Unico de Saúde* or SUS). Created in 1988, the SUS was conceived of as a national health service designed to provide comprehensive and universal care through decentralized management and provision of health services that are free of charge at the point of delivery. As of 2010 the SUS contained over 41,000 health posts and centers, 30,000 specialized outpatient services, nearly 2,000 public hospitals, and 236,000 community health agents [4]. In 2009 the SUS financed nearly 12 million hospitalizations and delivered about 100 million ambulatory care procedures per month [4]. In addition to the SUS, about 26% of Brazilian citizens have private health plans that allow them to access the private health sector in addition to the SUS.

While the SUS has expanded, there have been major changes in the socioeconomic conditions of Brazilians in recent years. For example, the mean real household income per capita increased from about US$(PPP) 225 in 1998 to $372 in 2009 and extreme poverty (measured by US$ PPP 1.25/day) declined from nearly 16% in 1998 to 4.7% in 2009 [5]. These changes have been attributed to a number of factors, including economic growth and social policies (such as increased minimum wages and social assistance cash transfers) focused on aiding the poor [6]. Other social investments have led to decreased illiteracy rates, increased school attendance, a more than doubling of completion rates in primary, secondary, and tertiary schooling since 1995, and improvements in child health and nutrition [5,7].

In spite of expansion of the SUS, especially in the area of primary health care through the Family Health Strategy (FHS), there is still concern about the Brazilian health system’s ability to improve equity in healthcare access [8]. Previously identified barriers include geographic and social inequalities in health services supply and other determinants of health [9,10]. Healthcare financing is also an important issue. Currently, the total government share of total health spending is estimated at 45%, which represents less than 4% of GDP—an amount that is lower than that of most other countries with universal health systems [4].

The objective of this study is to assess trends in horizontal equity (defined as the principle that people with the same healthcare needs should have similar access to healthcare services) in the utilization of different types of health services during a period of major economic and social change in Brazil.

## Methods

Our data come from a series of cross-sectional household surveys known as the National Household Sample Surveys (*Pesquisa Nacional por Amostra de Domicilios* or PNAD in Portuguese) carried out by the Brazilian Institute for Geography and Statistics (IBGE). We use the three health-related supplements to the PNAD conducted in collaboration with the Ministry of Health in 1998, 2003, and 2008. The PNAD uses a three-stage complex probabilistic sample, and is representative of the national, regional, and state levels [11]. A total of 1.12 million individuals are included in the three surveys, which obtained data by means of face-to-face interviews and rely on self-report.

We employ measures of horizontal equity developed by Van Doorslaer, Wagstaff, and others [12,13]. These measures seeks to assess equity (fairness) in healthcare utilization by taking into account the fact that individuals have different health needs and that differences in health needs ought to translate into different demand for and use of health services. But once health needs are standardized across individuals, remaining utilization could be considered to be inequitable. The horizontal inequity index (HI) is used to operationalize this concept. It is defined as the difference between observed healthcare utilization and that which would be expected given the individual’s health needs [14].

We base our set of control variables representing healthcare needs and other non-need factors on guidelines developed by the World Bank [14]. Each individual’s need for healthcare is approximated by the following variables: sex/age categories (12 variables representing men and women aged 0–17, 18–34, 35–44, 45–64, 65–74, and 75 years and over, with males aged 0–17 as the reference category), self-rated health (measured as excellent/good/fair versus poor/very poor); any physical functioning limitation (any difficulty in toileting/feeding/bathing oneself, kneeling/stooping, walking up stairs, or walking 100 meters); previous medical diagnosis of any of the following conditions: arthritis, cancer, diabetes, bronchitis/asthma, hypertension, heart disease, kidney failure, depression, tuberculosis, cirrhosis, and/or tendinitis; and a measure of co-morbidity (two or more of the conditions listed previously). Additional determinants of healthcare utilization (also known as non-need factors) include literacy (whether the person can read and write), schooling (less than 3 completed years, 4–7 years, 8–10,11-14, and 15 years or more), log monthly family income, urban/rural location, geographic region (North, Northeast, South, Southeast, Central-West), affiliation with a private health plan, and coverage by the Family Health Strategy (available only in 2008). For all control variables (except income) dummy variables were created for all categories, using the lowest category as the reference group. Note that we do not adjust monthly family income for inflation, since in the statistical analyses, income is used to rank each individual at each year along the income distribution and is treated as a relative measure of social position in each time period.

Outcome variables are measures of access and utilization of healthcare services that are comparable across the three surveys. These include: any doctor visit in the past 12 months, any dental care visit in the past 12 months, any hospitalization in the past 12 months, and any health services sought in the previous 2 weeks. An additional variable captures whether the individual is able to identify a usual source of medical care (“Do you tend to seek healthcare services from the same place?”) and is used a proxy measure of continuity of care.

Analysis of equity requires a series of steps. First, it is necessary to regress medical care utilization, *y*_*i*_ on a set of explanatory variables:

(1) $$y_{i}=\alpha +\beta \ln({\text{inc}}_{\text{i}})+\sum_{\text{k}}\gamma_{\text{k}}X_{{\text{k}}_{1}}+\sum_{p}\delta_{p}Z_{p_{1}}+\in_{i}\text{,}$$

where y_*i*_ is use of the particular health care service by individual *i,* ln(inc_i_) is the log of family income for individual *i*, X_k_ is a vector of need determining variables, Z_p_ is a vector of non-need determining variables, α, β, γ_k_ and δ_p_ are parameters and ϵ_i_ is the error term. This equation can be used to generate the predicted demand for any particular health care service,${\hat{y}}_{i}^{x}$**,** that is, the expected healthcare use of individual *i* on the basis of his/her health needs. This quantity can be thought of as the amount of the health care the individual should consume, if s/he had been treated the same as others with the same healthcare needs.

After predicting demand we calculate indirectly standardized demand (${\hat{y}}_{i}^{1\text{S}}$) by estimating the predicted y values by standardizing for the X (health need) variables while simultaneously controlling for the Z (non-need) variables.

(2) $${\hat{y}}_{i}^{x}=\hat{\alpha }+\hat{\beta }\ln\left(\bar{inc}\right)+\sum_{\text{k}}{\hat{\gamma }}_{\text{k}}X_{{\text{k}}_{1}}+\sum_{p}{\hat{\delta }}_{p}{\bar{Z}}_{p}$$

We then calculate ${\hat{y}}_{i}^{1\text{S}}={\text{y}}_{\text{i}}-{\hat{\text{y}}}_{i}^{x}+\bar{\text{y}}$, where ${\hat{y}}_{i}^{1\text{S}}$ is the indirectly standardized (predicted) demand, *y*_*i*_ is actual demand, ${\hat{y}}_{i}^{x}$ is the x-expected demand and y¯ is the sample mean of actual demand (See equation 2).

After completing the above standardization and graphing the concentration curves, we calculated the concentration index for both *y*_*i*_ and ${\hat{y}}_{i}^{1\text{S}}$, using the convenient regression method as outlined in O’Donnell et al [14]. Once the concentration indices for actual (C_m_) and predicted demand (C_p_) are calculated, the Horizontal Inequity Index (HI) is calculated as follows:

(3) $$HI=2\int_{0}^{1}\left[L_{p}(p)-L_{m}(p)\right]dp=C_{m}-C_{p}$$

where ***L***_***p***_***(p)*** is the concentration curve of predicted demand and ***L***_***m***_***(p)*** the concentration curve of actual demand. The HI ranges from −2 to 2 and is positive if there are inequities favoring the more advantaged members (richer) of society, which in these models is measured by family income.

Finally, we apply methods to decompose the concentration index in order to ascertain the contribution of each covariate (need and non-need factors as described above) to overall inequity in healthcare utilization. Because all outcomes are binary, we use techniques developed by Van Doorslaer et al [15-17]. Decomposition is performed using a linear approximation of the model based on partial effects of each covariate evaluated at the sample means. This approach allows us to identify which factors are associated with pro-rich or pro-poor utilization and to approximate their contribution to the overall concentration index.

Analyses were carried out using Stata Version 12 [18]. When appropriate, results are adjusted for the effect of the sample design and include individual probability weights.

## Results

Table 1 presents characteristics of the study sample in each survey year. Most variables have changed over the three survey periods. The mean age increased by about two years, although the male–female distribution did not change. Consistent with increased educational attainment in the country, the proportion of individuals with less than three years of schooling declined while those who completed 11 to 14 years nearly doubled. Literacy rates also increased significantly between 1998 2008. The process of urbanization increased over time although there was little change between 2003 and 2008. Monthly family income also rose, doubling from 1998 to 2008 (although part of this increase is due to inflation). Consequent with the aging of the population, the proportion of people in poor or very poor self-rated health, with one or more physical functioning limitation, with one or more chronic conditions, and those with two or more chronic conditions all increased significantly over time.

**Table 1:** Population Characteristics by survey year, Brazil 1998, 2003, 2008

	**1998**	**2003**	**2008**
Age (mean)	28.42	29.76†	31.67†‡
Female (%)	51.02	51.22	51.34
Schooling <3 years (%)	46.21	39.67†	34.78†‡
4-7 years (%)	27.61	26.57	23.91†‡
8-10 years (%)	11.58	13.28†	14.58†‡
11-14 years (%)	11.05	16.16†	20.90†‡
>14 years (%)	3.55	4.32†	5.84†‡
Literate (%)	75.68	79.08†	82.17†‡
Urban residence (%)	79.57	84.26†	83.75†
Mean family income (reais)	906.09	1207.19†	1904.30†‡
Private health plan (%)	24.45	24.46	25.89†‡
Poor/very poor self-rated health (%)	20.89	21.42†	22.72†‡
Covered by the Family Health Strategy (FHS) (%)	n/a	n/a	50.93
One or more physical functioning limitation (%)	7.04	7.17	8.51†‡
At least one chronic condition (%)	31.61	29.89†	31.32‡
Two or more chronic conditions (%)	14.0	12.38†	13.14†
At least one doctor visit in past 12 months (%)	54.69	62.82†	67.68†‡
Hospitalized at least once in the past 12 months (%)	6.94	7.01	7.11
Has a usual source of care (%)	71.22	79.27†	73.64†‡
Any health services use in the past 2 weeks (%)	12.99	14.59†	14.50†
At least one dental visit in the past 12 months (%)	33.15	38.74†	40.20†‡
Total n (unweighted)	344,975	384,834	391,868

All results take into account the complex sample design and include survey weights.

†Statistically significant difference (p <0.05) from 1998; ‡ statistically significant difference (p <0.05) from 2003.

Data source: Brazilian National Household Sample Survey (PNAD) 1998, 2003, 2008.

In terms of health services utilization there was a significant increase in the proportion of individuals who had at least one doctor visit in the past 12 months, although the proportion of the population hospitalized at least one time did not change. The proportion of the population who sought health services in the past two weeks increased between 1998 in 2003 but did not change between 2003 and 2008. There was an increase in the use of dental services in the past 12 months observed in each time period.

Table 2 shows the concentration indices (CI) representing the distribution of healthcare use across the income gradient in each year. The variable with the highest unadjusted positive CI (i.e. highest pro-rich orientation) is dental visits, while hospitalizations are the only negative values (i.e. pro-poor). Each measure also changes over time. The magnitude of the change of the CI varies between 51% reduction in the pro-rich orientation of doctor visits to a 625% change in having a usual source of care (USC). For each variable, the corresponding values for the HI are slightly larger in magnitude than those of the unadjusted CI, suggesting that pro-rich inequities are higher in each time period once healthcare needs are taken into account. Trends in changes to the HI over time are of the same sign and magnitude as those of the CI. All changes in the CI and HI between time periods are statistically significant (p <0.05) except for healthcare service use in the past two weeks between 2003 and 2008, and hospitalization between 2003 and 2008, both of which remained stable.

**Table 2:** Unstandardized Concentration Index (CI) and Horizontal inequity index (HI), by outcome and year

**Variable**	**Unstandardized Concentration Index (CI)**				**Horizontal inequity index (HI)**			
	**1998**	**2003**	**2008**	**% change**	**1998**	**2003**	**2008**	**% change**
				**1998 to 2008**				**1998 to 2008**
Doctor visit (12 months)	0.0500 (0.0011)	0.0400 (0.0021)	0.0330 (0.0016)	−51.52	0.0642 (0.0011)	0.0444 (0.0021)	0.0357 (0.0018)	−79.83
Hospitalization (12 months)	−0.0810 (0.0043)	−0.0530 (0.0051)	−0.0430 (0.0051)	88.37	−0.0430 (0.0043)	−0.0263 (0.0049)	−0.0127 (0.0049)	238.58
Usual source of care	0.0290 (0.0010)	0.0070 (0.0025)	0.0040 (0.0021)	−625.00	0.0323 (0.0010)	0.0079 (0.0025)	0.0039 (0.0029)	−728.21
Any healthcare service-use (2 weeks)	0.0660 (0.0032)	0.0440 (0.0047)	0.0440 (0.0042)	−50.00	0.1019 (0.0032)	0.0651 (0.0047)	0.0648 (0.0045)	−57.25
Dental visit (12 months)	0.2180 (0.0019)	0.1780 (0.0032)	0.1390 (0.0028)	−56.83	0.2308 (0.0018)	0.1943 (0.0032)	0.1514 (0.0029)	−52.44

Numbers are concentration indices with standard errors in parentheses.

Values <0 indicate pro-poor and >0 indicate pro-rich utilization.

HI is the difference between actual healthcare use and predicted utilization based on health needs.

All values are statistically significantly different from previous periods (p <0.05); except any healthcare service use for 2003 and 2008; and hospitalizations between 2003 and 2008.

Data source: Brazilian National Household Sample Survey (PNAD) 1998, 2003, 2008.

**Figure 1:**
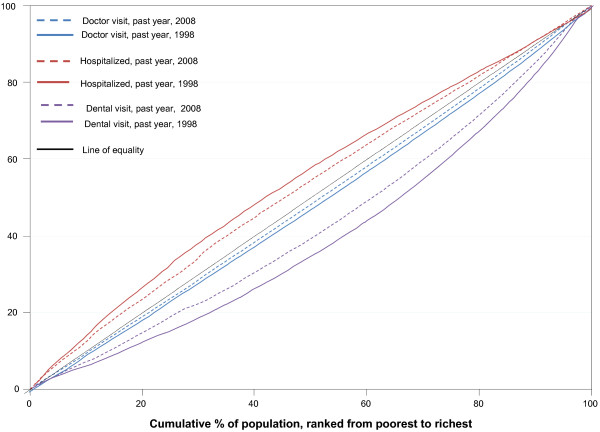
Lorenz curves for main healthcare utilization variables, 1998 versus 2008.

Figure 1 displays Lorenz Curves which illustrate the unadjusted concentration indices presented in table 2 for the main healthcare utilization variables in 1998 and 2008 across the entire income distribution. The figure shows that the distribution of hospital use, by income, was highly pro-poor in 1998 (below the line of equality). This pro-poor orientation declined in 2008, but still favored the poor. Doctor visits, on the other hand, were slightly pro-rich in 1998, but their income-related distribution moved closer to the line of income-equality in 2008. Dental visits displayed a high pro-rich orientation in 1998, moved closer to the line of income-equality each year, but still displayed a strong pro-rich orientation in 2008.

**Figure 2:**
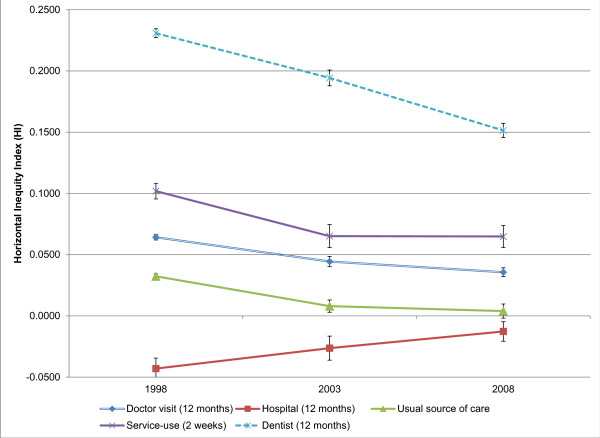
**Trends in the Horizontal Inequity Index (HI), 1998–2008, Brazil.** Note: The HI measure horizontal inequity. Positive values reflect pro-rich orientation, zero is perfect equivalence, and negative values represent an overall pro-poor orientation. Bars represent 95% confidence intervals. Data source: Brazilian National Household Sample Survey (PNAD) 1998, 2003, 2008.

Figure 2 shows trends in health inequality indices (HI) for each outcome. These trends are similar to those observed in the unadjusted concentration indices for most variables. Dental visits show the largest absolute decrease in horizontal inequity, although they are still the most inequitably (pro-rich) distributed service in 2008. Service use in the past two weeks shows decreased inequity in 2003 and no significant change in 2008. Doctor visits show a substantial decrease in 2003 and a smaller decrease in 2008, ending with a slightly pro-rich orientation. Having a usual source of care becomes less pro-rich over time and is nearly income-neutral by 2008. Hospital use is pro-poor in both 1998 and 2003 and becomes slightly less so by 2008.

In order to illustrate which factors have contributed to increased or decreased inequity for each outcome at each point in time, Table 3 presents decompositions of the concentration index into its component parts. The table demonstrates that the overall contribution of all need-related factors (as reported in the sub-total row) is negative or pro-poor. However, the contribution of the two main components of need factors (age and sex, and health problems) contribute in slightly different ways to different outcomes. Health problems contribute substantially to the pro-poor orientation of doctor visits, hospitalization, and health services sought in the past two weeks, while they are neutral or slightly pro-rich for dental visits and a usual source of care, as would be expected. Age and sex are generally negative, except in the case of doctor visits and hospitalizations, where they are either neutral or slightly pro-rich.

**Table 3:** Decomposition of the Concentration index, by outcome and year

	**Doctor visit (12 months)**			**Dental visit (12 months)**			**Service use (2 weeks)**			**Hospitalized (12 months)**			**Usual source of care**		
	**1998**	**2003**	**2008**	**1998**	**2003**	**2008**	**1998**	**2003**	**2008**	**1998**	**2003**	**2008**	**1998**	**2003**	**2008**
Need factors															
Age/sex	0.001	0.000	0.001	−0.012	−0.017	−0.015	−0.003	−0.004	−0.004	0.004	0.000	−0.003	−0.001	−0.001	−0.001
Health	−0.012	−0.002	−0.002	0.001	0.003	0.002	−0.030	−0.014	−0.016	−0.030	−0.017	−0.018	−0.002	0.000	0.000
Subtotal	−0.011	−0.002	−0.001	−0.011	−0.014	−0.012	−0.033	−0.018	−0.020	−0.025	−0.017	−0.022	−0.002	−0.001	0.000
Non-need															
Income	0.016	0.009	0.009	0.121	0.097	0.075	0.025	0.003	0.007	−0.072	−0.053	−0.040	0.002	−0.009	−0.004
Insurance	0.048	0.037	0.029	0.044	0.040	0.046	0.065	0.058	0.056	0.057	0.054	0.052	0.014	0.006	0.014
Schooling	−0.019	−0.014	−0.007	0.071	0.055	0.042	−0.018	−0.020	−0.011	−0.022	−0.025	−0.016	−0.008	−0.008	−0.009
Geography	0.020	0.014	0.009	0.011	0.015	0.008	0.029	0.023	0.027	0.011	0.004	0.005	0.023	0.017	0.012
FHS	-	-	−0.004	-	-	−0.012	-	-	−0.012	-	-	−0.010	-	-	−0.011
Subtotal	0.065	0.045	0.036	0.247	0.207	0.159	0.100	0.064	0.065	−0.026	−0.020	−0.010	0.031	0.006	0.003

Numbers represent each set of variables’ contribution to the concentration index. Positive values reflect pro-rich orientation, zero is perfect equivalence, and negative values represent an overall pro-poor orientation. Residuals not shown.

Age/sex represent all age and sex combinations. Health includes measures of poor self-rated health, physical functioning limitations, chronic conditions and comorbidity. Schooling includes being literate and years of schooling completed. Geography includes urban/rural and geographic region. FHS = Family Health. Strategy (measure only available in 2008).

Data source: Brazilian National Household Sample Survey (PNAD) 1998, 2003, 2008.

Non-need factors generally contribute to the pro-rich orientation of all variables, except hospitalizations. The largest single contributor to pro-rich orientation is the presence of a private health plan (health insurance). This is followed by the household’s geographic location and family income. Schooling generally contributes to the pro-poor orientation of healthcare services. Coverage by the Family Health Strategy (which is only present in the 2008 dataset) was a significant contributor to the pro-poor orientation of each outcome and its magnitude suggests it is an important contributor to the pro-poor orientation to each outcome in 2008.

When examining changes over time, the increased pro-poor orientation of most variables appears to derive from decreased pro-rich contributions of non-need factors and the additional effect of the family health strategy, since need factor contributions either stayed the same or became less pro-poor over time.

## Conclusions

Inequities in healthcare utilization are generally decreasing in Brazil. In 1998, they were already pro-poor for hospitalization, which represents the most costly and perhaps most urgent form of healthcare need. For medical care and dental visits, utilization of healthcare services is (to different extents) still pro-rich, although it has become increasingly less so over time.

One potential criticism of measures such as the HI is that they may be difficult to interpret. For this reason, we compare results with other studies using a similar set of methods. Among 10 OECD countries, Van Doorslaer et al [15] using data from the early 1990s, found that the HI measure (standardized using similar measures of health need including age, sex, self-rated health, and chronic conditions) for any doctor visit varied from 0.047 in the Netherlands to −0.010 in Germany, and for any hospitalization ranged from −0.076 in Denmark to −0.047 in Switzerland [15]. These results suggest that as of 2008, Brazil achieved an HI for all doctor visits close to that of Sweden (0.034) and slightly more pro-poor (closer to 0) than Denmark, Finland, the Netherlands, or the US in the 1990’s. For hospitalizations, Brazil’s level of inequity was more pro-poor than Switzerland and the United States.

Results differ slightly when compared to non-European countries using more recent data. For example, Lu et al [19] found that in 2002 the HI for doctor visits (0.093) and hospitalizations (0.064) to be more inequitable (pro-rich) in Hong Kong than we have found for Brazil. However, the same study found both HI measures (−0.009 for medical visits and −0.074 for hospitalizations) were considerably more pro-poor in South Korea than those reported here for Brazil [19].

The trends towards lesser horizontal inequity may reflect government policies around improving access to health care throughout the country. This includes expansion of the Family Health Strategy, which now covers over 50% of the Brazilian population with primary care services free of charge. The FHS (originally developed in the mid 1990’s) delivers comprehensive primary care services through a team of professionals that include a doctor, a nurse and 4–6 community health agents. The expansion of the FHS began with many smaller, less urban and poorer municipalities and within larger municipalities, priority for FHS expansion has often been in the poorest geographic regions, with some municipalities explicitly targeting more vulnerable regions for FHS implantation and expansion over time [20]. The FHS has also added over 20,000 oral health teams, which provide free dental care to people in 85% of Brazilian municipalities [21]. The accelerated expansion of the FHS during the 2000’s and its targeting towards more vulnerable populations may explain its association with more pro-poor access and use of healthcare and dental services within the country.

Changes in hospitalizations are likely to reflect changes in supply within the Brazilian health system and shifts in the profile of conditions for which people are hospitalized [22]. The lack of changes in service use in the past two weeks may be due to the fact that the measure asks about any healthcare service use. Previous studies have shown that visits to primary care providers (general practitioners) are generally more pro-poor than visits to specialists [17]. Since the early 2000’s there have been increases in supply of both primary care and some forms of specialist and diagnostic services, and this may have resulted in increased utilization by the poor being offset by increased utilization by the rich among these different types of services.

Results show that the main contributors to pro-rich healthcare inequities in Brazil are non-need factors, most importantly, income, geographic location, and the presence of a private health plan. This difference may be explained by different patterns of utilization among those using the public versus the private sector for healthcare services, with higher utilization for nearly all services among those with a private health plan. The need-standardization techniques used here show that utilization (especially among the rich) appears to be above and beyond what would be expected given their health needs. Overutilization of healthcare services may be undesirable because of increased healthcare costs with limited marginal benefits and potential iatrogenic effects of unnecessary tests and procedures [23]. Higher than expected rates of utilization among the poor may also be problematic. In the case of Brazil, higher rates of hospitalization among the poor may represent lack of access to some preventive services or the use of hospitals as a substitute for primary care.

This study has several limitations. First, all utilization data are based on self-report, but since the questions were asked in nearly identical formats in all three years, this preserves comparability. One exception is self-report of chronic diseases, since in 1998 respondents were asked if they had any of a list of chronic conditions, while in 2003 and 2008 the survey specified “medically-diagnosed” self-report. Another potential limitation is the fact that we use family income instead of other economic measures such as household wealth. This choice of this measure was based on the fact that the PNAD surveys are the gold standard in Brazil for measuring household and family income. One disadvantage is that this survey may have underestimated the extent of extreme wealth present in Brazil, although this is a common limitation of many national household surveys [24].

Finally, it is important to note that greater equity in access to and utilization of health services does not immediately translate into greater equity in health outcomes. Although some aspects of equity in health (particularly for children) have shown advances, such improvements are generally thought to be as much a result of changes to socioeconomic conditions as to access to appropriate health services [25].

In conclusion, these results suggest that inequities in healthcare utilization in Brazil are not as pronounced as might be expected, given lingering social inequalities within the country. The trend toward greater horizontal equity is a potential indicator of success in government efforts to improve access to care, especially among the poor. In order to continue on this trajectory, the Brazilian health system will need to continue to address areas where there may be under-utilization (such as in yearly dental visits), but also assess potential unnecessary over utilization of care. One strategy currently being pursued is the strengthening of primary health care. Based on the results presented here, that strategy, amongst others, may have already contributed to making healthcare utilization more equitable in Brazil.

## Competing interests

The authors declare they have no competing interests.

## Authors’ contributions

JM and MFLC conceived the study. JM carried out data analysis and drafted the text. MFLC helped to draft and revise the manuscript and interpreted results. Both authors read and approved the final manuscript.
