# Erratum: Horizontal equity in health care utilization in Brazil, 1998-2008

**DOI:** 10.1186/s12939-014-0101-7

**Published:** 2014-11-13

**Authors:** James Macinko, Maria Fernanda Lima-Costa

**Affiliations:** Department of Nutrition, Food Studies & Public Health, New York University, 411 Lafayette Street, 5th Floor, New York, NY 10003 USA; Centro de Pesquisas René Rachou, Fundação Oswaldo Cruz and School of Medicine, Federal University of Minas Gerais, Av. Augusto de Lima 1715, Belo Horizonte, CEP: 30190-002 MG Brazil

## Erratum

While the article entitled Horizontal equity in health care utilization in Brazil, 1998–2008 [1] was being prepared for publication, a typographical error was introduced in the methods section. The sentence “The HI ranges from −**2 to 2** and is positive if there are inequities favoring the more advantaged members (richer) of society, which in these models is measured by family income”. should have read: “The HI ranges from −**1 to 1** and is positive if there are inequities favoring the more advantaged members (richer) of society, which in these models is measured by family income”.
